# New insights on intercontinental origins of paternal lineages in Northeast Brazil

**DOI:** 10.1186/s12862-020-1579-9

**Published:** 2020-01-29

**Authors:** Ana Paula Schaan, Leonor Gusmão, Juliana Jannuzzi, Antonio Modesto, Marcos Amador, Diego Marques, Silvia Helena Rabenhorst, Raquel Montenegro, Thayson Lopes, France Keiko Yoshioka, Giovanny Pinto, Sidney Santos, Lorenna Costa, Vivian Silbiger, Ândrea Ribeiro-dos-Santos

**Affiliations:** 1 Human and Medical Genetics Laboratory, Federal University of Pará, Av. Augusto Corrêa, 01 – Cidade Universitária Prof. José Silveira Netto - Guamá, Belém, PA 66075-110 Brazil; 2 DNA Diagnostic Laboratory (LDD), Institute of Biology, State University of Rio de Janeiro (UERJ), Rio de Janeiro, Brazil; 3 Center for Oncology Research, Federal University of Pará, Belém, PA 66073-005 Brazil; 4 Pathology and Legal Medicine Department, Federal University of Ceará, Fortaleza, CE 60020-181 Brazil; 5 Genetics and Molecular Biology Laboratory, Federal University of Piauí, Parnaíba, PI 64202-020 Brazil; 6 Clinical and Toxicological Analyses Department, Federal University of Rio Grande do Norte, Natal, RN 59300-000 Brazil

**Keywords:** Y-SNPs, Population genetics, Genetic ancestry, Asymmetric colonization, Admixed population

## Abstract

**Background:**

The current Brazilian population is the product of centuries of admixture between intercontinental founding groups. Although previous results have revealed a heterogeneous distribution of mitochondrial lineages in the Northeast region, the most targeted by foreign settlers during the sixteenth century, little is known about the paternal ancestry of this particular population. Considering historical records have documented a series of territorial invasions in the Northeast by various European populations, we aimed to characterize the male lineages found in Brazilian individuals in order to discover to what extent these migrations have influenced the present-day gene pool. Our approach consisted of employing four hierarchical multiplex assays for the investigation of 45 unique event polymorphisms in the non-recombining portion of the Y-chromosome of 280 unrelated men from several Northeast Brazilian states.

**Results:**

Primary multiplex results allowed the identification of six major haplogroups, four of which were screened for downstream SNPs and enabled the observation of 19 additional lineages. Results reveal a majority of Western European haplogroups, among which R1b-S116* was the most common (63.9%), corroborating historical records of colonizations by Iberian populations. Nonetheless, *F*_*ST*_ genetic distances show similarities between Northeast Brazil and several other European populations, indicating multiple origins of settlers. Regarding Native American ancestry, our findings confirm a strong sexual bias against such haplogroups, which represented only 2.5% of individuals, highly contrasting previous results for maternal lineages. Furthermore, we document the presence of several Middle Eastern and African haplogroups, supporting a complex historical formation of this population and highlighting its uniqueness among other Brazilian regions.

**Conclusions:**

We performed a comprehensive analysis of the major Y-chromosome lineages that form the most dynamic migratory region from the Brazilian colonial period. This evidence suggests that the ongoing entry of European, Middle Eastern, and African males in the Brazilian Northeast, since at least 500 years, was significantly responsible for the present-day genetic architecture of this population.

## Background

Present-day Brazilians are the result of centuries of admixture between three main ethnic groups, namely Native Americans, Europeans and Africans (1). As the first colonizers of the American continent, Amerindians arrived via Bering strait and reached Brazilian territory during the late Pleistocene (2). Later, the European component of the Brazilian colonization had an important impact on the composition of the current day populations.

Besides Portuguese settlers, other European groups also invaded or populated the country, such as Spanish, Dutch, French, English, Italian, German, Middle-Eastern and Japanese populations. Most of these settlements initially took place in the Northeastern and Southern regions. In fact, the Northeast was the most targeted area for migratory events during the colonial period and received the greatest number of European and African individuals (1, 3–5). Therefore, admixture processes between distinct ethnic groups began much earlier and intensively in the Northeast than in the rest of the country, which makes this an important region for investigating Brazil’s demographic history.

Uniparental markers are useful for disentangling the complex processes that shaped the current population (6). Indeed, previous data from maternal and paternal lineages (using both slow and fast evolving markers) has shown a strong male biased colonization of the Brazilian territory, with the majority of mitochondrial DNA (mtDNA) haplogroups being of Native American and African origin, while Y-chromosome lineages are overwhelmingly dominated by European haplogroups (7–14). We have demonstrated heterogenous frequencies of mitochondrial Amerindian and African lineages in the Brazilian Northeast, which brought a new perspective to the understanding of the maternal ancestral contributions to this region (13). An insight into the paternal lineages of this same population is important to determine whether male contributions were also distinct from earlier reports and to what extent the numerous historical immigrations influenced the genetic architecture of this region (7, 9).

Given its non-recombining nature and low mutation rates, single nucleotide polymorphisms (SNPs) located in the Y-chromosome are a useful tool for investigating historical events and serve as a valuable counterpart for our mtDNA data (15–17). The geographic specificity enabled by this marker allows for an even greater characterization of the microevolutionary aspects of Brazil’s present-day population (6).

**Fig. 1:**
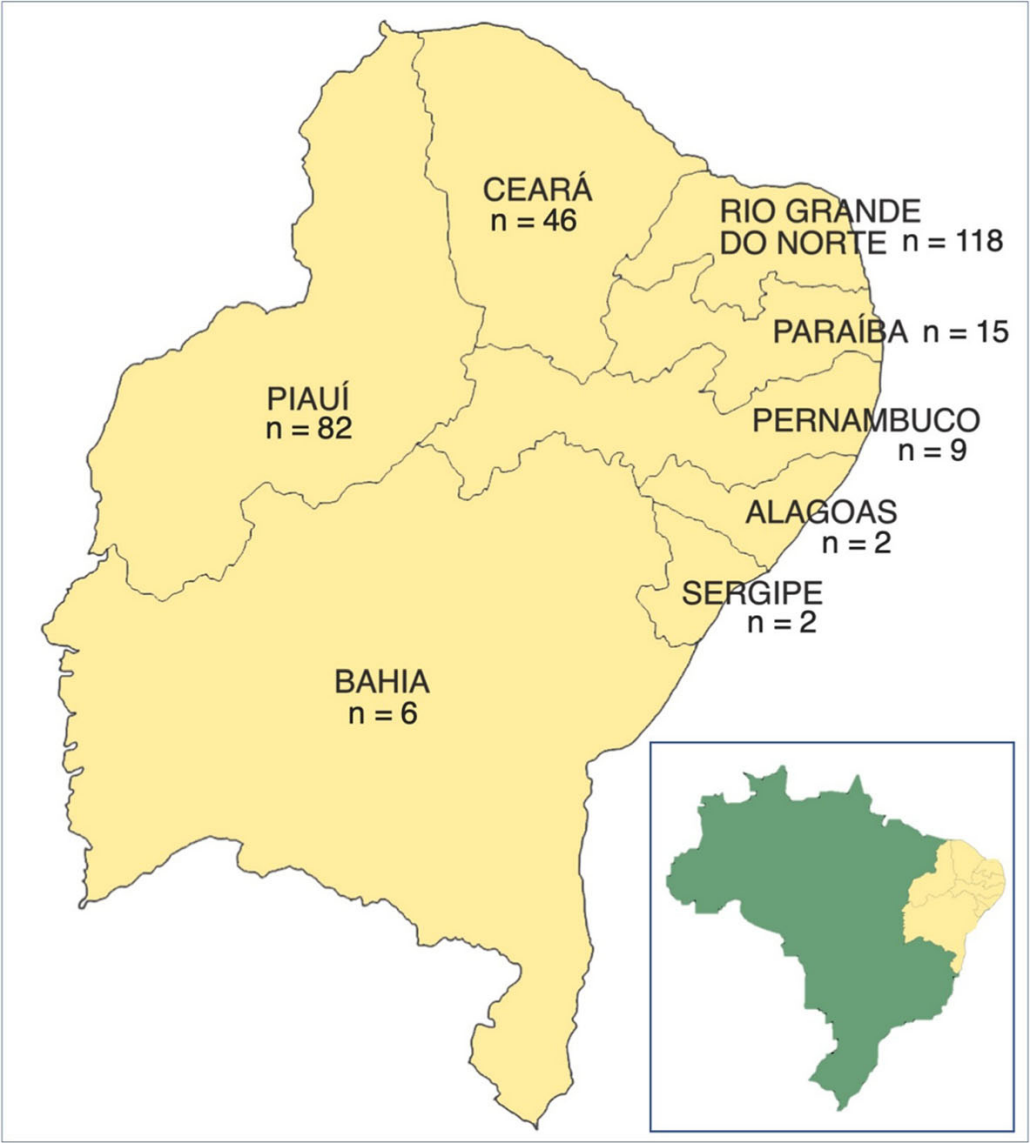
Map and sample sizes of the investigated regions in this study. MA – Maranhão, PI – Piauí, CE – Ceará, RN – Rio Grande do Norte, PB – Paraíba, PE – Pernambuco, AL – Alagoas, SE – Sergipe, BA – Bahia. Created with Adobe Photoshop CC 2019 (www.adobe.com/products/photoshop.html)

**Fig. 2:**
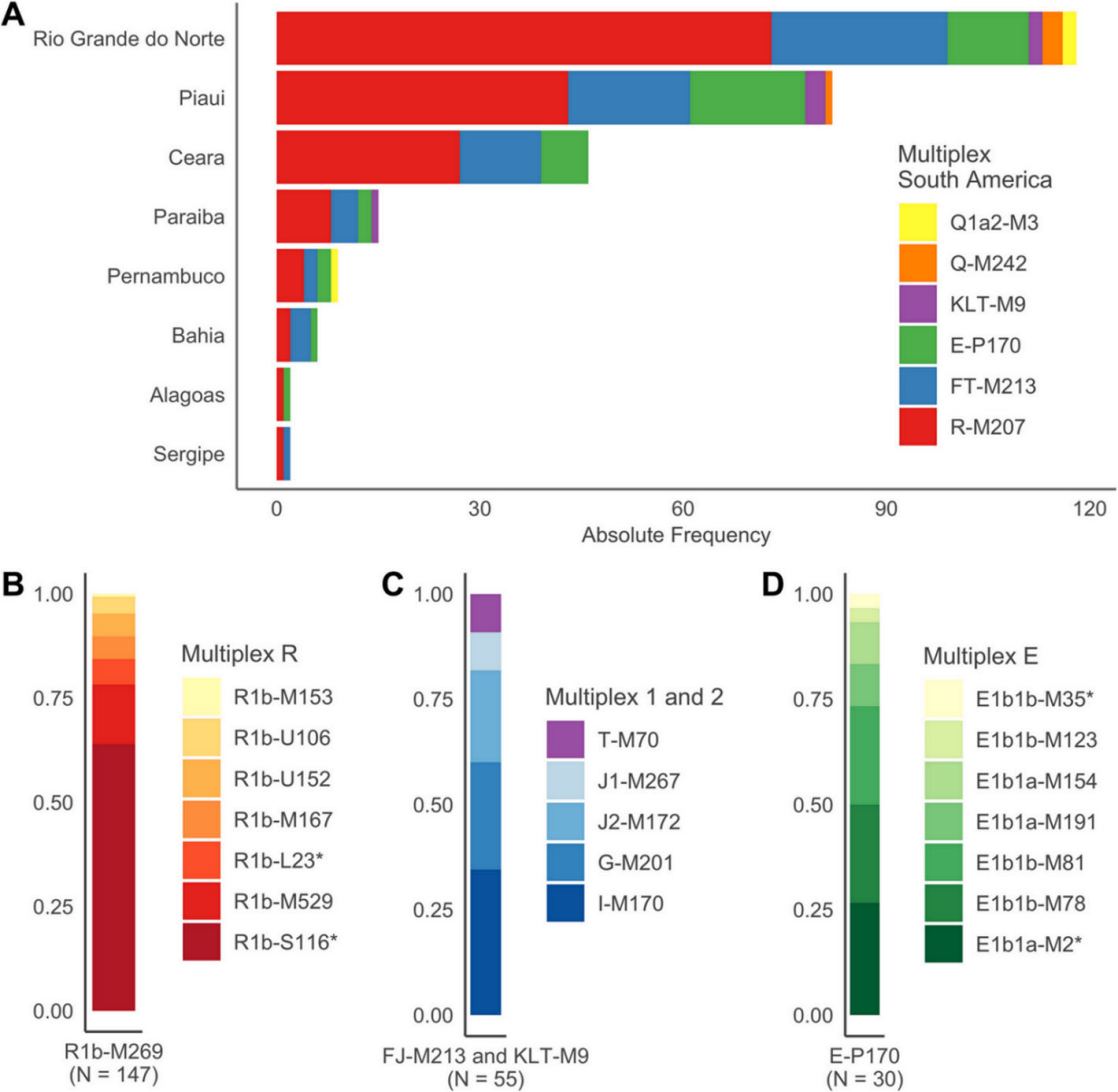
Y-Chromosome phylogeny of the SNPs investigated here through five Multiplex reactions

In this paper, a sample of 280 unrelated male individuals from several Northeastern states is analysed in a hierarchical assay for determining Y-chromosomal ancestral lineages (Fig. 1). Four subsequent high-resolution multiplex assays were also carried out to better characterize the haplogroups present in the Northeast region of Brazil (Fig. 2). Additionally, data was compared to previously obtained mitochondrial lineages from the same samples in order to provide a comprehensive description of the ancestral background and demographic history of this particular population.

## Results

### Y-chromosome Haplogroups in the northeastern Brazilian population

Data obtained from the Major South America Multiplex is shown in Table 1. All genotypes and relevant data regarding the SNPs and population chosen in this study are available in Additional file 1. In the Northeastern region, the majority of samples belonged to the R-M207 haplogroup (56.8%), followed by FJ-M213 (23.6%), E-P170 (15%), KLT-M9 (2.1%), Q-M242 (1.4%) and Q1a2-M3 (1.1%). This pattern is representative of all states surveyed in this study (with *N* ≥ 15), showing that there is a homogenous distribution of Y-chromosome haplogroups in the investigated locations (Pearson’s Chi-Square test, *p = 0.6606*).

**Table 1:** Results obtained from Multiplex Major South America

Population	*N*	Haplogroup frequencies					
		E-P170	FJ-M213	KLT-M9	Q-M242	Q1a2-M3	R-M207
Northeast	280	0.150	0.236	0.021	0.014	0.011	0.568
Piauí	82	0.207	0.220	0.037	0.012	–	0.524
Ceará	46	0.152	0.261	–	–	–	0.587
Rio Grande do Norte	118	0.102	0.220	0.017	0.025	0.017	0.619
Paraíba	15	0.133	0.267	0.067	–	–	0.533
Pernambuco	9	0.222	0.222	–	–	0.111	0.444
Alagoas	2	0.5	–	–	–	–	0.5
Sergipe	2	–	0.5	–	–	–	0.5
Bahia	6	0.167	0.500	–	–	–	0.333

*n* Sample size

**Fig. 3:**
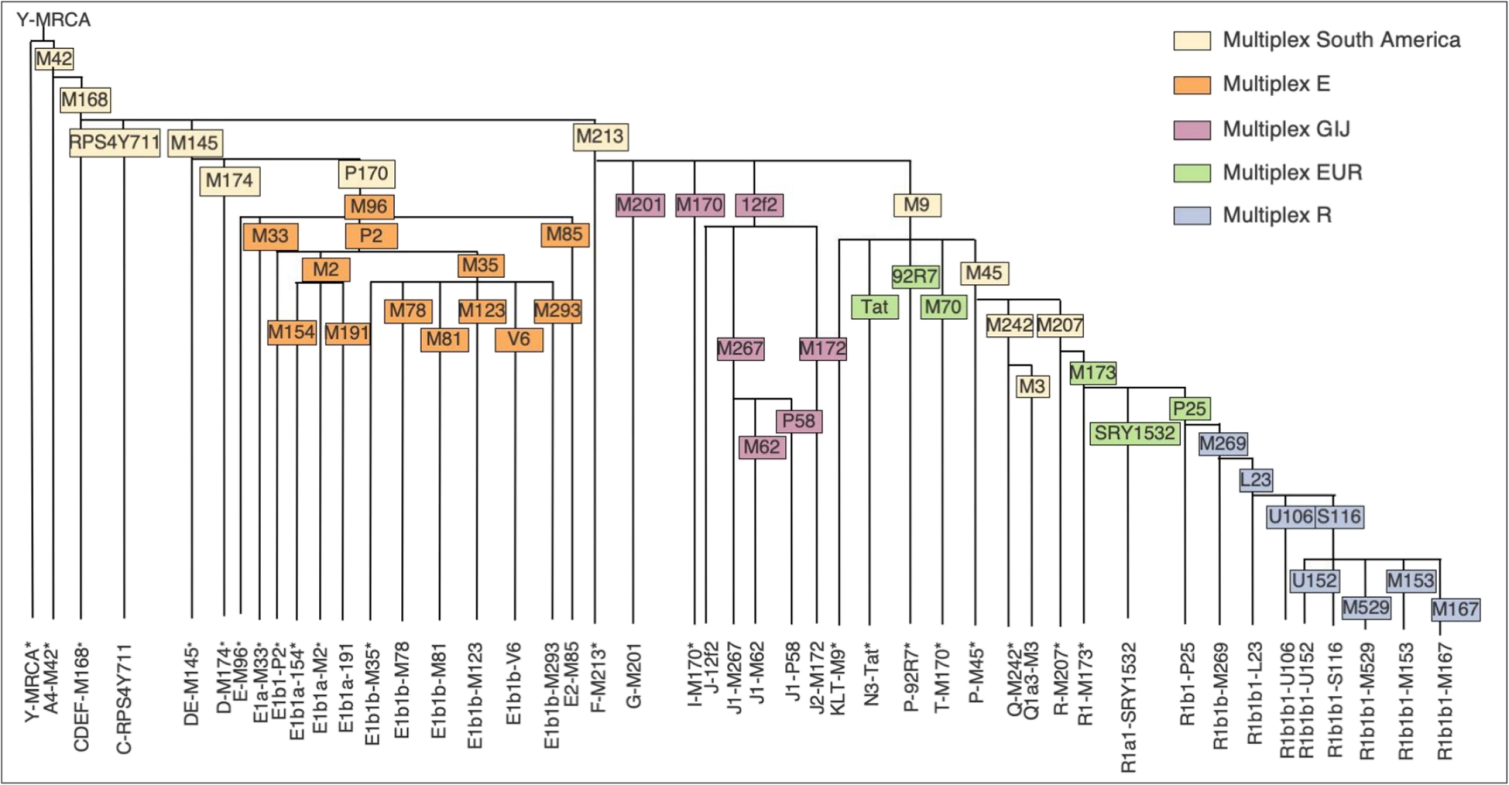
Frequency of Y-Chromosome Haplogroups in Northeastern Brazil

Considering the globally widespread presence of certain Y-chromosome haplogroups, we performed the investigation of 33 downstream SNPs through multiple Multiplex reactions in order to provide higher phylogenetic resolution of the paternal ancestry composition of this population (Table 2, Fig. 3).

**Table 2:** Diversity and relative frequencies of Y-SNP haplogroups found in eight Northeastern Brazilian states

Haplogroups	Population								
	Northeast	Piauí	Ceará	Rio Grande do Norte	Paraíba	Pernambuco	Alagoas	Bahia	Sergipe
E1b1b-M123	0.004	0.017	–	–	–	–	–	–	–
E1b1a-M154	0.013	0.017	–	–	0.067	–	–	0.167	–
E-M2*	0.033	0.068	0.024	0.019	–	–	–	–	–
E-M35*	0.004	–	0.024	–	–	–	–	–	–
E1b1b-M78	0.029	0.017	0.073	0.019	0.067	–	–	–	–
E1b1b-M81	0.029	0.034	0.024	0.028	–	–	0.500	–	–
E1b1a-M191	0.013	0.034	–	0.009	–	–	–	–	–
R-M153	0.004	–	0.024	–	–	–	–	–	–
R-M167	0.033	0.017	–	0.047	–	0.125	–	0.167	–
R1b-L23*	0.038	0.068	0.024	0.028	–	0.125	–	–	–
R1b-M529	0.088	0.051	0.024	0.132	0.133	0.125	–	–	–
R1b-S116*	0.393	0.492	0.366	0.377	0.400	0.125	0.500	0.167	0.500
R1b-U106	0.025	0.034	0.049	0.019	–	–	–	–	–
R1b-U152	0.033	–	0.122	0.028	–	–	–	–	–
I-M170	0.079	0.051	0.049	0.104	0.133	0.125	–	–	–
Q-M242	0.017	0.017	–	0.028	–	–	–	–	–
Q-M3	0.013	–	–	0.019	–	0.125	–	–	–
G-M201	0.059	0.051	0.098	0.047	0.067	–	–	0.167	–
T-M70	0.021	0.034	–	0.019	0.067	–	–	–	–
J1-M267	0.021	–	0.049	0.028	–	–	–	–	–
J2-M172	0.050	–	0.049	0.047	0.067	0.125	–	0.333	0.500
***N***	239	59	41	106	15	8	2	6	2
*HD ± s.d.*	1.000 ± 0.0003	0.8440± 0.0322	0.8744 ± 0.0391	0.8559 ± 0.0256	0.8381 ± 0.0852	1.000 ± 0.0524	1.000 ± 0.5000	0.9333 ± 0.1217	1.000 ± 0.5000

*N* Number of subtyped samples; *k* Number of different haplogroups; *HD* Haplotype diversity; *Belongs to the clade but not a subclade

### Sublineages of R1b-M269 haplogroup

To address the lack of data regarding European lineages in many states from Northeastern Brazil, we carried out the sub-typing of eight R1b-M269 downstream SNPs in 147 samples with the R-M207 derived allele (14). This methodology allowed the detection of lineages R1b-S116*, R1b-M529, R1b-U152, R1b-U106, R1b-L23*, R1b-M167, and R1b-M153. Sub-types derived from the M269 marker have been shown to be the most frequent in Western Europe, which make them useful for determining the origin of Brazilian patrilineages (18–21).

R1b-S116* was the most common haplogroup in this subset of samples, corresponding to 63.9% of individuals, while R1b-M529 showed a frequency of 14.3% as the second most frequent. Haplogroup R1b-L23* was observed in 6.1% of samples, R1b-M167 and R1b-U152 shared a frequency of 5.4%, R1b-U106 was assigned to 4.1% of samples, and lastly, R1b-M153 was the least frequent, accounting for 0.7% of the subjects.

**Fig. 4:**
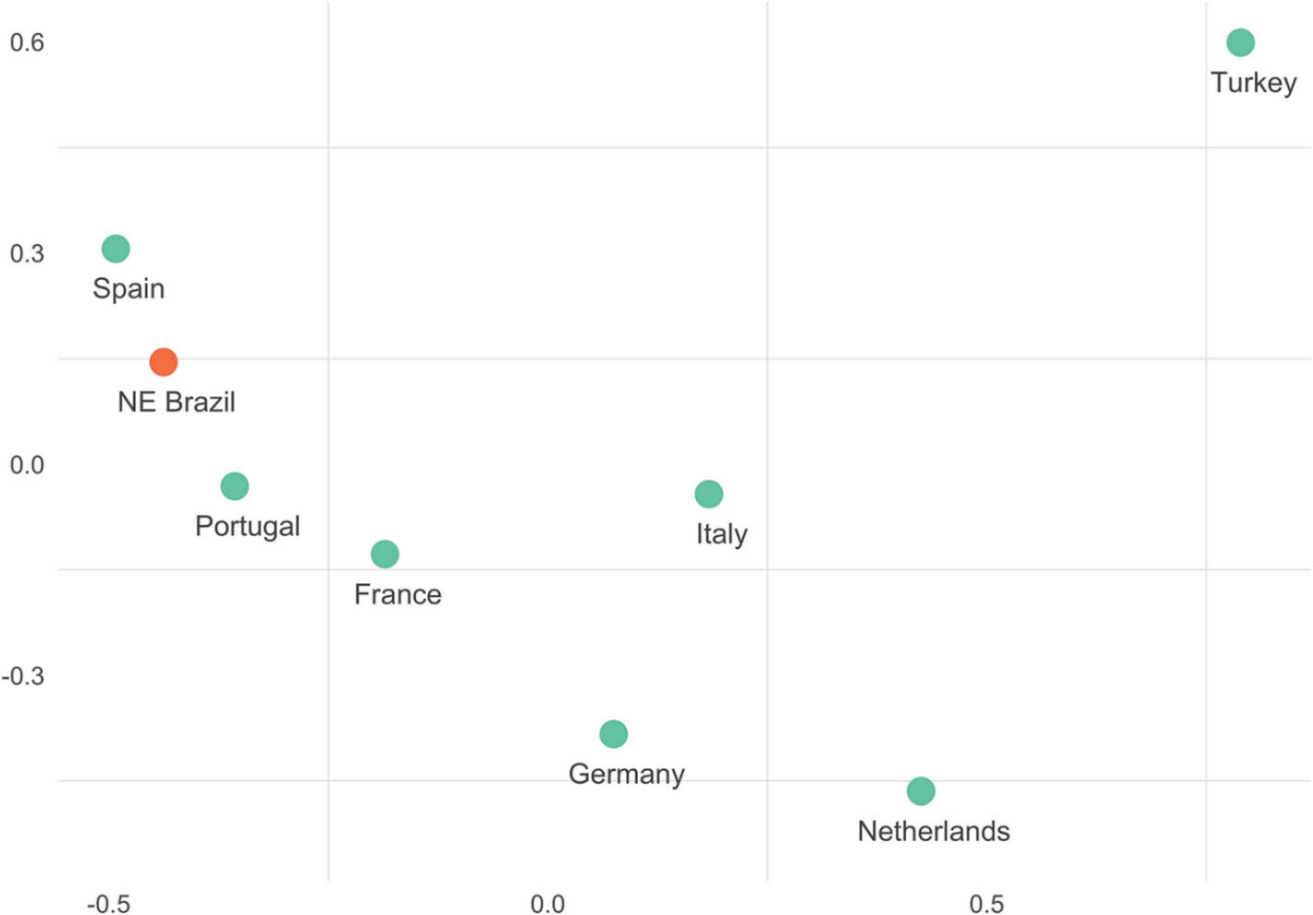
Multidimensional scaling plot of the pairwise *F*_ST_ genetic distances based on the frequencies of R-L23*, R-U106, R-S116*, R-U152 and R-M529 haplogroups in Northeastern Brazil and in seven European populations that contributed to the formation of the Brazilian gene pool. Stress = 4.139478e-14

To test whether it was possible to observe diverse sources of European lineages in the Northeast, we computed *F*_ST_ genetic distances based on R1b-M269* sub-type frequencies. A multidimensional scaling plot demonstrates the distances between the Brazilian population investigated here and previous data obtained from potential European colonizer populations (19, 22) (Fig. 4). The Brazilian sample is closest to populations from the Iberian Peninsula, while still showing some proximity to other Western European populations.

### Sub-lineages of FJ-M213 and KLT-M9 haplogroups

In order to further investigate the European genepool of Brazil’s Northeastern population, it was necessary to consider sub-lineages derived from deep-rooting markers FJ-M213 and KLT-M9. These haplogroups contain lineages that are mostly present in current day Europeans, Middle Eastern and Near Eastern populations (23).

Accounting for 23.6% of individuals investigated in this study, 50 samples belonging to haplogroup FJ-M213 were genotyped for six downstream SNPs, which allowed the detection of four sub-lineages. Of these, sub-haplogroup I-M170 was the most frequent (38%), followed by G-M201 (28%), J2-M172 (24%), and J1-M267 (10%). Additionally, Multiplex 1 was performed on five samples assigned to haplogroup KLT-M9, all of which belonged to rare sub-haplogroup T-M70, typically attributed to Shepardic populations (24).

### Sub-lineages of E-P170 haplogroup

A set of 13 downstream SNPs was chosen for increased resolution of 30 samples carrying the E-P170 marker. The importance of determining the ancestral origin of E haplogroup sub-lineages relies in the multiple colonization processes that took place in Northeast Brazil, which not only included the forced migration of many Sub-Saharan African groups, but also included subsequent North African and Middle Eastern immigrants (3).

Multiplex E results show that all samples belonged to subclades of the P2 marker, E-M35 and E-M2. The former, observed in 53.3% of these individuals, included E1b1b-M35*, E1b1b-M81, E1b1b-M78, and E1b1b-M123. From the latter, E1b1a-M2*, E1b1a-M191, and E1b1a-M154 represented 46.7% of samples.

## Discussion

The homogenous distribution of European haplogroups in Northeastern Brazil showed by our results is expected in this region of the country due to historical reports and previous local data (7, 9). A greater European component of paternal lineages is also the case for the other geopolitical regions of Brazil, which is a demonstration of the colonization patterns in this territory (7, 10, 11, 14, 25–27). The investigation of subtypes of these deep-rooted haplogroups was important for elucidating the origin of foreign settlers that have historically contributed to the formation of the Brazilian gene pool, given that historical records describe intense migratory movements of diverse populations in this region since the colonial period (1).

The most frequent haplogroup in our sample, R-M207*, is the most commonly observed in Europe, indicating the origin of more than 50% of Northeast Brazilian lineages (19, 22, 28–30). Regarding sub-lineages derived from the M269 marker, S116* was the most common in our sample, which is in agreement with previous findings from the Northeastern states of Alagoas and Maranhão (9, 31). This haplogroups is also the most common in the Iberian peninsula, corroborating the historical occupation of Northeastern Brazil by men of Portuguese origin (32, 33). This haplogroup was followed by R1b-M529 as the second most common, which can be found at high frequencies in England and Ireland (34).

Findings for the state of Ceará (14) and Maranhão (31) show lineage R1b-M529 at 2.2 and 3% frequencies, respectively, in accordance with our findings for Ceará (2.4%). However, for the state of Rio Grande do Norte, this contribution is five times larger, making up 13.2% of the population. Interestingly, this same state has higher frequencies of haplogroup I-M170 (10.4%) when compared to most states investigated in this study. Considering both of these haplogroups are rarely found in western Europe, these results may reveal the continued presence of non-Iberian colonizers in Northeast Brazil, corroborating historical reports (1, 5).

Another example of differential haplogroup distribution in this region that may reflect historical occupations is the case of R1b-U152, which was observed four times more frequently in Ceará (12.2%) than in Rio Grande do Norte and Maranhão (31). This haplogroup is currently mostly found in Northern Italy, France and Germany (19).

Regarding the remaining M269 derived lineages found in frequencies ranging from 2.8–0.4% in our sample, R1b-U106 has been reported as most frequent in Northwestern Europe, R1b-M153 and M167 were reported in Iberian populations and their descendants, mostly in the Basques (19, 35–38), and R1b-L23* reaches its maximum frequency in the Balkans, Turkey, the Caucasus and the Circum-Uralic region (19). Our results are further substantiated by data from Carvalho-Silva (7), who described the interesting heterogeneity of European male haplogroups in the Northeastern region (which were also found to be common in the South) and brought attention to the fact that this region was largely inhabited by the Dutch during the seventeenth century. Therefore, the presence of multiple European lineages in this population is in agreement with historical records from the period of such settlements.

Indications of multiple colonizer sources are also observed through the presence of haplogroups G-M201, J-M267, and J-M172, common in the Middle East and Near Eastern regions (23, 39). These haplogroups have shown to be distributed in diverse frequencies throughout the Northeastern territory, with G-M201, for instance, varying from 11% in Bahia (40) to 3.6% in Alagoas (9). According to Resque et al. (14), the presence of these lineages is possibly a product of the immigration of Arab traders in the post-colonial period.

Such discussion may be further extended to the presence of other Middle Eastern and North African haplogroups E-M78 and E-M81 in our findings, both present in 2.9% of individuals. One should consider that similar frequencies are found in Iberian populations, meaning this contribution could be from a European colonizer source (33). However, it is worth noting that both haplogroups were found at a frequency of 8% in the state of Maranhão (31), suggesting they may indeed originate from North African and/or Middle Eastern groups. Data from that same study also shows that over 30% of Maranhão individuals have African haplogroups, potentially supporting a non-European origin for the aforementioned haplogroups in the Brazilian Northeast.

With regards to E haplogroups in our findings, the presence of M2 derived markers, which is restricted to Sub-Saharan Africa, seems to be a product of the transatlantic slave trade, responsible for the arrival of West African individuals mostly to the Northeast region of Brazil during the seventeenth century. The contribution of haplogroups E-M2*, E1b1a-M191, and E1b1a-M154 show that the ancestral background of these Brazilian men is derived from a signature of the Bantu expansion, which is consistent with prior studies (14, 41). Interestingly, a whole genome sequence investigation performed by Kehdy et al. (42) for the population of Salvador (capital of Bahia) yielded signatures of both Bantu and non-bantu genetic ancestry, indicating a greater complexity to the African background of Northeastern Brazilian men.

Finally, the least frequent haplogroups in our sample, accounting for a total of 1.4%, are derived from the Q-M242* polymorphism, which is confined to the American continent and Amerindian populations (43–45). Such small contributions of African and, ever more so, of Native American haplogroups are in accordance with Y-chromosome data from other European colonies in South America, despite them being the majority in mtDNA studies (46–49). In fact, previous mtDNA data obtained by Schaan et al. (13) for the same samples demonstrated a strong Amerindian (43.5%) and African (37.8%) female component in Northeast Brazil. These findings attest to the strong asymmetric colonization favoring the introgression of European Y lineages in this region, a pattern that has been reported for other Brazilian regions and is typical for South American countries as well (50).

## Conclusions

In conclusion, our data brings biological evidence to historical records stating the importance of intercontinental arrivals to Northeast Brazil since the colonial period. Through the analyses of 45 Y-chromosome SNPs, we demonstrated that Iberian ancestry is represented in the majority of individuals. Still, the presence of other non-western European lineages is a strong indicator of the continued presence of multiple historically relevant occupations. Furthermore, the frequency of Middle Eastern haplogroups may suggest more recent immigrations, while common African Bantu lineages probably reflect the transatlantic slave trade. Overall, these results reveal the complex structure of the ancestral male genetic background of the Brazilian Northeast, and contribute to the knowledge of South American demographic history.

## Methods

### Population sample and DNA extraction

We tested a total of 280 unrelated male samples from the Northeastern region of Brazil, distributed in eight states as follows: i) 82 from Piauí, ii) 46 from Ceará; iii) 118 from Rio Grande do Norte; iv) 15 from Paraíba; v) nine from Pernambuco; vi) two from Alagoas; vii) two from Sergipe; viii) and six from Bahia, as shown in Fig. 1. These samples are a subset of those previously investigated for mtDNA data. See Schaan et al. (13) for biological material acquirement and DNA extraction methodology.

### Genotyping

In total, 45 SNPs were analysed in this work (Fig. 2). SNP typing was performed through multiplex polymerase chain reactions (PCR) and Single Base Extension (SBE) analysis using the SNaPshot kit (Thermo Fisher Scientific, Waltham, MA.) The data obtained in this study is available in Additional file 1. For determining population substructure based on the main ethnic groups that compose the Brazilian gene pool, 12 SNPs were chosen based on the hierarchical Multiplex Major South America assay described by Geppert et al. (51). This initial screening allowed for the identification of five lineages, namely haplogroups from the E-P170, FJ-M213, KLT-M9, Q-M242, and Q1a2-M3 branches. Samples were subsequently genotyped according to obtained results, consisting in: i) Multiplex 1, for samples carrying derived allele M9 (Brion et al., 2004); ii) Multiplex GIJ, for samples with derived allele M213 (52); iii) Multiplex E, for samples with derived allele E-P170 (53); and iv) Multiplex R, for samples with derived allele R-M207 (14).

### Statistical analysis

Haplogroup frequencies were determined by direct counting. Population genetics parameters such as comparisons, diversity values and population pairwise genetic distances (*F*_ST_) were computed using the Arlequin software v.3.5.2.2 (54). *F*_ST_ values were visualized in a multi-dimensional scaling (MDS) analysis and haplogroup frequency distribution analysis (Pearson’s Chi-squared test) were performed on R software v.3.5.3 (55). Haplogroup frequencies of samples carrying the M269* derived allele were compared to those found in current European populations and data was extracted from Myres et al. and Busby et al. (19, 22). For this purpose, we also included the 41 R1b-M269 derived samples from Ceará tested by Resque et al. (14) into our analysis.

### Availability of data and material

All data generated or analysed during this study are included in the supplementary information files.

## Supplementary information

**Additional file 1.** Genotypic and SNP data. All genotypes found for the samples analysed in this study as well as chosen SNP information can be found in Additional file 1.

## Data Availability

All data generated or analysed during this study are included in this published article [and its supplementary information files].

## Abbreviations

mtDNA: Mitochondrial DNA
PCR: Polymerase chain reaction
SNP: Single nucleotide polymorphism
